# Mechanism and application of *Lactobacillus* in type 2 diabetes-associated periodontitis

**DOI:** 10.3389/fpubh.2023.1248518

**Published:** 2023-11-30

**Authors:** Sisi Chen, Yuhan Zhang

**Affiliations:** ^1^Chongqing Three Gorges Medical College, Chongqing, China; ^2^Chongqing Medical University, Chongqing, China

**Keywords:** periodontitis, type II diabetes, *Lactobacillus*, mechanism, application

## Abstract

Type 2 diabetes mellitus (T2DM) accelerates the progression of periodontitis through diverse pathways. Abnormal immune responses, excessive activation of inflammation, increased levels of advanced glycation end products, and oxidative stress have defined roles in the pathophysiological process of T2DM-associated periodontitis. Furthermore, in the periodontium of diabetic individuals, there are high levels of advanced glycation end-products and glucose. Meanwhile, progress in microbiomics has revealed that dysbacteriosis caused by T2DM also contributes to the progression of periodontitis. *Lactobacillus*, owing to its fine-tuning function in the local microbiota, has sparked tremendous interest in this field. Accumulating research on *Lactobacillus* has detailed its beneficial role in both diabetes and oral diseases. In this study, we summarize the newly discovered mechanisms underlying *Lactobacillus*-mediated improvement of T2DM-associated periodontitis and propose the application of *Lactobacillus* in the clinic.

## 1 Introduction

Periodontitis is a destructive chronic inflammatory disease characterized by lumen swelling, hemorrhage, auxiliary bone absorption, and loose teeth (1). Type 2 diabetes mellitus (T2DM), one of the most common chronic diseases, is often accompanied by systemic complications and is an independent risk factor for periodontitis. Meanwhile, numerous studies provide evidence of the reciprocal interactions between T2DM and periodontitis. Moderate to severe periodontitis has been reported to increase the risk of T2DM and lead to poor glycemic control in diabetic patients (2, 3). Moreover, the elevation in circulating IL-6, TNF-a, and CRP levels caused by periodontitis contributes to increased systemic inflammation, thus further aggravating insulin resistance in patients with T2DM (4). Correspondingly, diabetes increases the morbidity of periodontitis and accelerates the progression of periodontitis (5). The activation of inflammatory and oxidative stress signaling pathways, increased levels of advanced glycosylation end products (AGEs), and abnormal immune responses have been confirmed to play a role in diabetes-related periodontitis (6). In recent years, with the development of microbiomics, the role of dysbiosis caused by T2DM in periodontal disease has attracted attention. The increase in *Porphyromonas gingivalis* (*P. gingivalis*), *Prevotella intermedia* (*P. intermedia*), and other harmful bacteria and their metabolites associated with inflammation and insulin resistance in the gingiva causes periodontal tissue damage (7, 8). Therefore, interventions targeting the dysbiosis in the oral flora can improve the conditions of T2DM and periodontitis (9, 10).

Probiotics are “living microorganisms,” as defined by the World Health Organization and the Food and Agriculture Organization. Probiotics live in the human body and exert their beneficial effects (11) by suppressing the growth of pathogenic microorganisms, producing bioactive metabolites, and maintaining the balance of the local microenvironment (12). Among all defined probiotics, *Lactobacillus* is the most studied commercial probiotic. The first commercial *Lactobacillus* species was *Limosilactobacillus reuteri* (*L. reuteri*) (13). Since then, more strains have become commercially available, and they are heterofermentative *Lactobacillus*, such as *Lactiplantibacillus plantarum* (*L. plantarum*), *Lacticaseibacillus rhamnosus* (*L. rhamnosus*), *Limosilactobacillus fermentum*, *Lactobacillus acidophilus* (*L. acidophilus*), and *Ligilactobacillus salivarius* (*L. salivarius*) (14). These strains have been widely applied in acute diarrhea, cardiovascular disease (15), genitourinary tract infection (16), cancer (17), food allergy (18), Crohn’s disease (19), pouch inflammation (19), and colitis (20). More recently, *Lactobacillus* was found to be an immune regulator that activates lymphocytes in the gastrointestinal tract after colonizing the intestinal mucosa (21). Studies have also recognized the role of these strains in alleviating lactose intolerance and decreasing antibiotic-related side effects. *Lactobacillus* has also been extensively used in oral diseases such as ozostomia and caries due to its antibacterial effect (22). Regarding periodontitis, local application of *Lactobacillus* improved periodontitis directly by reducing the depth of periodontal pockets, alleviating gingival bleeding, and suppressing alveolar bone resorption (23).

The beneficial roles of *Lactobacillus* in T2DM have been widely defined. *L. rhamnosus* GG (LGG) has been shown to reduce the incidence of gestational diabetes (24). Another *Lactobacillus* species—*L. acidophilus* has been demonstrated to lower blood glucose in patients with T2DM (25). These results sparked tremendous interest and prompted us to explore the underlying mechanisms. The improvement of glucose metabolism, the removal of excess reactive oxygen species, relief of the inflammatory state, and regulation of the gut microbiota have all been shown to be potential mechanisms by which *Lactobacillus* improves T2DM, and these mechanisms are also involved in the treatment of periodontitis.

Recent intensive studies have revealed the complex regulatory network through which *Lactobacillus* impacts T2DM-associated periodontitis. Periodontitis is no longer attributed to an infection by a single or a few bacterial species. In essence, periodontitis is now classified as a dysbiotic disease, which arises as a result of the feed-forward loop involving polymicrobial communities and a dysregulated host inflammatory response. During this process, the beneficial role of *Lactobacillus* in regulating the immune system, oxidative stress and microbiota, antibacterial activity, and glucose metabolism helps delay the process of periodontitis. These mechanisms also contribute to improving blood glucose control in T2DM. In this article, we review the newly discovered pathogenic mechanisms and applications of *Lactobacillus* in patients with type 2 diabetic periodontitis; as such, we hope to provide a new and viable option for the clinical treatment of T2DM-associated periodontitis.

## 2 Related mechanisms

### 2.1 Reducing inflammation and regulating immunity

#### 2.1.1 Regulating the immune response and cytokines

A possible underlying mechanism by which *Lactobacillus* improves T2DM-associated periodontitis is by promoting a beneficial host response and reducing adverse changes in periodontal tissue. Studies have shown that the probing pocket depth of patients with periodontal disease was significantly reduced following *Lactobacillus* treatment, which may be due to the immunomodulation by *Lactobacillus* that leads to anti-inflammatory effects (26). *P. gingivalis* induces the synthesis of proinflammatory mediators (such as TNF-α and IL-1β) by activating TLR-4. *L. rhamnosus* lr-32; *L. acidophilus* La-5 can adhere to human gingival epithelial cells (GECs) and downregulate the expression of TLR-4, therefore reducing the adhesion of pathogens and their invasion (27). In addition, *P. gingivalis* coordinates the host response by circumventing the defense mechanism triggered by gingival epithelial cells, thus expanding the wound area in periodontitis patients. The expression of CXCL8, a key factor that regulates the proliferation/migration of epithelial cells, can be upregulated by *Lactobacillus* (28). *Lactobacillus* can also accelerate re-epithelialization by upregulating the expression of members of the CXCL8-CXCR1/CXCR2 axis, thereby reducing or reversing the harmful effects of *P. gingivalis* infection and improving wound healing in periodontitis (29). In addition to CXCL8, *Lactobacillus* can also regulate re-epithelialization through indirect antagonism by inhibiting pathogen adhesion. Euterin and reutericyclin, derived from *L. reuteri,* are antibacterial substances that inhibit a wide range of pathogens by inducing oxidative stress in cells and preventing the blinding of peripheral pathogens to host tissue (30).

The balance between activated matrix metalloproteinases (MMPs) and their inhibitors (TIMPs) controls the degree of extracellular matrix (ECM) remodeling (31). During T2DM-associated periodontitis, the imbalance between activated MMP and TIMPs leads to the pathological destruction of the ECM (32, 33). Among MMPs, MMP-8 is the most abundant collagen-soluble MMP, which contributes to tissue destruction and remodeling in patients with periodontitis. Studies have shown that MMP-8 abundance is closely related to the severity of periodontal disease, and *Lactobacillus* reduces MMP-8 in gingival crevicular fluid (34). Additionally, MMP-9 is related to host defense mechanisms, and studies have shown that MMP-9 levels are increased and TIMP-1 levels are decreased in patients taking LGG.

Periodontitis is a common chronic inflammatory disease that is characterized by disordered glucose metabolism and the cytokines: interleukin-1 (IL-1), interleukin-6 (IL-6), tumor necrosis factor-α (TNF-α), and interleukin-17A (IL-17A) (35). Inflammatory byproducts and bacterial endotoxins and metabolites are the main causes of periodontal damage; of these, IL-6 is important for alveolar bone resorption. Accordingly, T2DM is recognized as a chronic, systemic, and low inflammation state. Experimental evidence from animals and humans shows that inflammation is critical to the induction of insulin resistance in obese individuals. Activated macrophages in adipose tissue are responsible for inflammation. Cytokines associated with blood sugar control include TNF-α, IL-6, and IL-10, which may have insulin receptor substrates (IRSs) that are converted to serine, which leads to insulin resistance. Therefore, the regulation of cytokines (reducing the production of anti-inflammatory cytokines and increasing the production of anti-inflammatory cytokines) is of crucial importance to improving T2DM-associated periodontitis. Many experiments have shown that cytokine levels can be altered by *Lactobacillus*.

Inflammation is mainly caused by bacterial components such as lipopolysaccharide (LPS), which is the primary component of the Gram-negative endotoxin extracellular membrane (26). Ketones produced by *Lactobacillus* through the polyunsaturated fatty acid (PUFA) pathway exert anti-inflammatory functions via mitogen-activated protein kinase (MAPK) and NF-κB signaling in LPS-induced macrophages; furthermore, the resulting ketophilic acid produced also suppresses the production of IL-6, IL-1β, and TNF-α (36). Various types of *Lactobacillus* reduced the severity of T2DM, thereby improving the condition of T2DM-associated periodontitis. Huang et al. confirmed that mixed therapy with *L. plantarum* K68 and FVF controlled the increase in IL-1β, IL-6, and TNF-α in insulin-resistant mice (37). *Lacticaseibacillus casei (L. casei)* inhibited macrophage production of TNF-α, reduced the level of TNF-α, IL-1β, and IL-6, and increased short-chain fatty acid (SCFA)-producing intestinal hyperlumaccharide-1 (GLP-1) levels (38). Additionally, further studies showed that other *Lactobacillus* strains also have similar effects. *L. rhamnosus*, *L. acidophilus, L. brevis,* and *L. reuteri* reduce the expression of TNF-α and increase the level of IL-10 (39). Increased IL-10 can downregulate the expression of proinflammatory cytokines such as IFN-γ and IL-2/IL-1β, thereby preventing T2DM-associated periodontitis. *Lactobacillus* also inhibits the progression of chronic periodontitis by inhibiting the secretory activity of Th17 lymphocytes, which are responsible for excessive cytokine responses in the pathogenesis of the disease and can lead to adverse changes in periodontal tissues (40). There are also reports that *Lactobacillus* can reduce the inflammatory state of other diseases.

#### 2.1.2 Maintaining intestinal barrier function

*Lactobacillus* may also improve T2DM-associated periodontitis by helping to maintain intestinal barrier function. Based on the hypothesis of the leaky epithelium, intestinal microbiome dysregulation leads to increased intestinal permeability, which allows bacterial endotoxins to enter circulation, impair intestinal barrier function, and eventually cause an immune response that damages β cells and may lead to an increase in cytokine secretion, thus causing insulin resistance (41). Previous studies have shown that *L. plantarum WCFS1* can induce the expression of genes related to the anti-inflammatory immune response, increase the immune response in the human intestinal tract (42), promote cell growth and proliferation, stimulate TLR2 as a regulator of epithelial integrity, and regulate the expression of epithelial tight junctions to help maintain the environmental balance of the intestine. Similarly, Zo-1 and occludin were repositioned near the tight junction following *L. plantarum WCFS1* administration, which affects intestinal barrier function (43). *L. rhamnosus* GG also reduced intestinal permeability in mice fed a high-fructose diet, which stimulated goblet cell production of mucin and prevented LPS and other pathogens from crossing the intestinal barrier (44).

#### 2.1.3 Disrupting bacterial biofilm

The formation of bacterial biofilms in the oral cavity is considered the main cause of many pathological conditions in the oral cavity, and periodontitis is not an exception. *Lactobacillus* may inhibit oral biofilm formation and reduce harmful inflammatory immune responses (45). *Lactobacillus* may also contribute to the regulation of periodontal immune inflammation by altering the composition of bacterial biofilms. The oral colonization of *Prevotella melaninogenica (P. melaninogenica)* is associated with periodontitis. *L. brevis* CD2 and *L. reuteri* inhibit melanin-producing *P. melaninogenica* biofilms. The possible mechanism is that *L. reuteri* inhibits the production of bacterial ribonucleotide reductase and has antibacterial effects (46). A high concentration of *L. acidophilus* CFF significantly inhibited biofilm formation, removed biofilms, and stimulated monocytes/macrophages. A more than 90% reduction in biofilm formation was achieved with the highest concentrations of *L. acidophilus* WCS and CFF (47).

Whether by regulating cytokines, maintaining intestinal barrier function to reduce inflammation, or removing biofilms and reducing periodontal tissue damage through host responses, we speculate that *Lactobacillus* has the potential to reduce inflammation and regulate immunity, thus reducing the severity of T2DM-associated periodontitis.

### 2.2 Regulating oxidative stress

Reactive oxygen species (ROS) are key components of the neutral granulocyte antibacterial library. However, excessive ROS often leads to oxidative stress in periodontal tissue with increasing production of proinflammatory cytokines, such as IL-6 and TNF-α, in gingival epithelial cells that induces pathological changes and leads to the destruction of the structure supporting host teeth and the loss of teeth (48). ROS also increases insulin resistance and impairs the β cell membrane, thereby promoting the occurrence and development of diabetes (49). Therefore, regulating oxidative stress may be an effective way to treat T2DM-associated periodontitis.

Many studies have confirmed that *Lactobacillus* strains have high antioxidant capacity. The antioxidant mechanism of *Lactobacillus* is closely related to the removal of reactive oxygen species and the increase in antioxidants (50). The metabolites of *L. plantarum,* ketoacid, and hyaluronic acid, stimulate the expression of antioxidant-related genes in gingival epithelial cells, inhibit the oxidation process, and prevent inflammation (51). Accordingly, *in vivo*, *L. plantarum* increased glutathione peroxidase activity in diabetic rats and upregulated the expression of the peroxisome proliferator-activated receptors-α (PPAR-α) and γ (PPAR-γ) to prevent oxidative stress that damages insulin-secreting cells and protect pancreatic function. *L. casei* and *Lacticaseibacillus paracasei* (*L. paracasei*) also have the ability to scavenge free radicals and antioxidant activity; they can also restore the level of malondialdehyde (MDA), a marker of oxidative stress (52, 53). In addition to the above *Lactobacillus* strains that reduce oxidative stress, several other *Lactobacillus* strains also exert antioxidant effects in other ways.

Excessive NO leads to barrier dysfunction, and high levels of NO promote the infiltration of inflammatory cells into periodontal tissues by inducing pathological vascular permeability (54). NO plays a role in periodontal inflammation and bone loss, as has been confirmed in animal models. Relevant evidence shows that *Lactobacillus* reduces oxidative stress by inhibiting NO production. *Lactobacillus* reduces the expression of the nitric oxide synthase (iNOS) gene in fat, restores the overall energy balance in animal adipose tissue, and inhibits the inflammatory response (55). Studies also show that *L. plantarum* reverses intestinal imbalance caused by diabetes, increases intestinal ROS levels, and reduces intestinal iNOS expression induced by diabetes (56). In addition, arginine deaminase, an enzyme that metabolizes arginine into citrulline and ammonia, is present in *L. brevis* and helps *L. brevis* extract inhibit NO production by competing with NOS for the same substrate, arginine (57). Harisa et al. (58) demonstrated that in diabetic rats, the level of NO returned to normal after applying oral *L. acidophilus*, perhaps by reducing oxidative stress; thus, there was an antidiabetic effect.

### 2.3 Improving glucose metabolism

Another underlying mechanism by which *Lactobacillus* treats T2DM-associated periodontitis is the regulation of glucose metabolism. A variety of probiotics have been identified to directly control or reverse the elevation of glycosylated hemoglobin and fasting insulin levels. There is a meta-analysis of 12 randomized controlled trials on the role of probiotics in T2DM, 10 of which clearly indicate that *Lactobacillus* does significantly reduce HbA1c, fasting insulin levels, and HOMA-IR levels in T2DM (59).

#### 2.3.1 Reducing glycogen synthesis and increasing glycogen consumption

Several investigations have shown that *Lactobacillus* may directly inhibit glucose synthesis and gluconeogenesis. For instance, *L. plantarum* CCFM0236, *L. casei* CCFM0412, and *L. acidophilus* SJLH001 increase α-glucosidase activity and reduce food intake, blood glucose level, and the glycosylated hemoglobin level; furthermore, *L. acidophilus* KLDS1.1003 and *L. acidophilus* KLDS1.0901 induced downregulation of the expression of glycogen synthase kinase in mice (60). There is more evidence showing that after the administration of *L. rhamnosus,* the mRNA expression of gluconeogenesis genes, especially glucose-6-phosphatase, in the gluconeogenesis pathway was inhibited (61). Probiotic-fermented milk prepared by *L. rhamnosus* MTCC: 5957, *L. rhamnosus* MTCC: 5897, and *L. fermentans* MTCC: 5898 significantly reduced the expression of key enzymes in the gluconeogenesis pathway (62). Dang F et al. applied *L. paracasei* TD062 to T2D mice and proved that it could downregulate the expression of gluconeogenic genes (52). In terms of glycogen consumption, probiotic-fermented milk containing *L. acidophilus* and *L. casei* has been shown to reduce FBG and HbA1c, increase muscle glucose uptake, and stimulate liver glucose absorption, which is consistent with the conclusions of another meta-analysis (59).

#### 2.3.2 Decreasing insulin resistance

Studies have shown that the increase in blood glucose levels in rats is often accompanied by severe insulin resistance (63), and many *Lactobacillus* strains have decreased insulin resistance, thereby reducing the blood glucose level in diabetic rats. For example, *L. casei CCFM419* reduce insulin resistance and hyperglycemia in T2D mice (38, 64). In diet-induced obesity model mice, *Lactobacillus* reduced insulin resistance and increased glucose tolerance, possibly by reducing endoplasmic reticulum stress in skeletal muscle, inhibiting macrophage activation, and promoting the transcription of glucose transporter 4 (65, 66).

Adiponectin (APN) is known as a fat factor that prevents the development of insulin resistance and T2DM. Increased serum APN is related to an increase in insulin sensitivity (67). Fibroblast growth factor 21 (FGF21) can promote the expression of adiponectin, and LGGs may alleviate PPAR-α through butyric acid activation, thereby effectively increasing the expression of FGF21 and the insulin sensitivity of mice (68). β cells also play an important role in blood glucose control; they can activate PI3K and Akt and regulate glycogen synthase (69) to control blood glucose. Matsuzaki et al. reported that *Lactobacillus* increased insulin binding in a T2DM mouse model and prevented cell destruction (70).

#### 2.3.3 Increasing insulin levels in the body

There is no doubt that insulin plays a crucial role in T2DM-associated periodontitis, and blood glucose instability is often accompanied by insufficient amounts of insulin. Evaluation of insulin status in rats revealed that *Lactobacillus* may increase insulin levels by increasing insulin synthesis and inhibiting insulin decomposition and consumption. Glucagon-like peptide 1 (GLP-1) is a key incretin-stimulating hormone that plays a major role in insulin secretion (71). Application of a specific *Lactobacillus* strain (*L. reuteri* SD5865) increased insulin secretion by increasing GLP-1 release of incretin (72). *Lactobacillus* can also stimulate insulin secretion by regulating autonomic neurotransmitters, inhibiting the expression and activity of insulin-degrading enzyme (IDE), and reducing insulin consumption to slow the decline in insulin and the rise of blood glucose levels (73).

As a special substance, SCFAs, which are organic fatty acids produced by bacterial fermentation in the distal intestine, can be quickly transferred to the blood system and regulate glucose metabolism by a variety of mechanisms (74). The mechanisms by which SCFAs regulate glucose homeostasis may include (1) promoting the proliferation of intestinal epithelial cells and helping to maintain the integrity of the intestinal barrier; (2) reducing gluconeogenesis and inhibiting glycogen decomposition; and (3) increasing GLP-1 secretion, β cell quality, and function; stimulating intestinal endocrine cells to secrete peptide YY (PYY) and glucagon-like peptide 1 (GLP-1); and accelerating glucose-mediated insulin secretion. *L. paracasei* HII01 *and L. casei* CCFM419 may improve hyperglycemia by inhibiting the SCFA pathway (63), which further shows that *Lactobacillus* can alter glucose metabolism in several ways.

In summary, T2DM-associated periodontitis is characterized by a glucose standard, so it is feasible for *Lactobacillus* to improve glucose metabolism in different ways, including lowering blood sugar levels directly (reducing glycogen synthesis and increasing glycogen consumption) or indirectly (decreasing insulin resistance and increasing insulin levels in the body).

### 2.4 Direct resistance to pathogens

Compared with non-diabetic subjects, diabetic subjects had higher levels of *Streptococcus sanguinis, Prevotella nigrescens, Treponema denticola, Streptococcus intermedius, and Streptococcus oralis* in their dental plaque (75). Studies have clearly shown that most *Lactobacillus* strains are capable of inhibiting *Actinomycetes*, *P. gingivalis*, *P. intermedia,* and *Streptococcus mutans* growth, with the strongest antimicrobial activity being seen with parthenogenic heterogeneous fermenting *Lactobacillus* (*L. plantarum*, *L. paracasei*, and *L. rhamnosus*) and *L. salivarius* (76). The possible underlying mechanisms by which *Lactobacillus* directly inhibits the growth of other bacteria are the secretion of antibacterial proteins called bacteriocins and the production of toxic metabolites such as hydrogen peroxide, which may be produced by enzymes such as pyruvate oxidase, lactate oxidase, NADH oxidase, and NADH-independent reductase in *Lactobacillus*. Bacteriocins produced by *Lactobacillus* include the salivaricin produced by *L. salivarius*, the reuterin produced by *L. reuteri*, and the phytotoxin produced by *L. plantarum* (23). Bacteriocin forms short-lived pores on biofilms by interacting with different compounds, such as nisin, lipid II, and phosphorus, to eliminate pathogens. *Lactobacillus* produces lactic acid, an effective microbicide to prevent the colonization of bacterial and viral pathogens, and hydrogen peroxide and bacteriocin, which play an antibacterial role against oral pathogens. For example, the specific strain *L. delbrueckii* can inhibit *P. gingivalis* growth *in vitro* through the autolytic release of protein, which produces hydrogen peroxide and reacts with the Fenton of iron in the cell to form reactive oxygen species (77), thereby damaging DNA and inhibiting cell activity. Accordingly, another study found that in microaerobic environments *in vivo*, such as the interface between gingival crevices and teeth, *L. delbrueckii* also produces hydrogen peroxide, and hydrogen peroxide can diffuse freely in close enough proximity to *P. gingivalis* to impact the growth of *P. gingivalis* (78).

In addition to directly reducing pathogens, downregulating virulence gene expression to treat T2DM-associated periodontitis is also a viable strategy. Pathogenic bacteria in dental plaque biofilms invade periodontal tissues and secrete toxins such as lipopolysaccharide to destroy the supporting structure of teeth, thus negatively impacting periodontal health. Heat-inactivated *L. acidophilus* has been proven to bind to HOK cells from the oral cavity, which is the first step in treating T2DM-associated periodontitis. Following coaggregation with heat-inactivated *Lactobacillus*, the expression of the virulence gene fap2 of *Fusarium nucleatum* was significantly reduced, and its self-aggregation, adhesion, and invasion were largely restricted; consequently, the expression of proinflammatory genes in oral epithelial cells triggered by *Fusarium nucleatum* was prevented (79).

Overall, in terms of the direct removal of pathogenic bacteria, high concentrations of *Lactobacillus* CFF can achieve more than 99.99% bacterial clearance. In terms of attenuating the expression of virulence genes, *Lactobacillus* strongly attenuates the transcription of *P. gingivalis* virulence genes, thus reducing their virulence (47). Moreover, the metabolite ketone of *Lactobacillus* significantly inhibited *P. gingivalis* both *in vivo* and *in vitro* by reducing alveolar bone damage and suppressing *P. gingivalis* activity, proliferation rate, and CT values (36).

### 2.5 Regulating the microbiota

#### 2.5.1 Regulating the oral microbiota

Periodontitis is a mixed anaerobic infection disease that may be in great part related to periodontal pathogens, including *P. gingivalis, P. intermedia*, *Treponema denticola,* and *Clostridium nucleatum* (80). *Lactobacillus* might modulate subgingival microflora to treat T2DM-associated periodontitis, which is mainly related to reducing the number of target pathogens through competitive colonization. *Lactobacillus* has good colonization potential (23) and is able to compete with *P. gingivalis* in terms of adhering to epithelial cells (27). A previous study showed that the level of pathogenic bacteria (especially *P. gingivalis*) in the periodontal pocket decreased after the application of *Lactobacillus*, such as *L. salivarius* WB21 (81). *Lactobacillus* may play a beneficial role in the oral cavity through direct and indirect interactions with microorganisms in the dental plaque. Lactic acid produced by *Lactobacillus* creates an acidic environment, which inhibits the growth of *Gingival actinomycetes, Intermediate actinomycetes,* and *Streptococcus mutans.* This effect may be the reason why *Lactobacillus* inhibits the growth of these microorganisms.

In addition to the above bacteria, the symbiosis of *Candida albicans* and *Actinomycetes* increases significantly in the periodontal environment; therefore, *Candida albicans* is also closely related to periodontitis (82). Thus, the inhibition of *Candida albicans* by *Lactobacillus* likely contributes to the balance of the oral microbiota. Animal studies have shown that *Lactobacillus* can reduce *Candida* infection, inhibit the growth of *Candida* by producing antibacterial compounds, and prevent its adhesion to epithelial cells through competitive colonization. In addition, in an *in vitro* model simulating gastrointestinal conditions, *Lactobacillus* also inhibited the growth of *Candida* (83), possibly by competing for the same receptor site.

#### 2.5.2 Regulating the gut microbiota

The role of periodontal-related bacteria in the development of T2DM-associated periodontitis is clearly documented by studies showing that periodontal pathogens lead to intestinal flora disorder by moving to the intestine in rat and mouse models (84). Accordingly, the intestinal microbiota of T2DM patients is disordered, with a decrease in butyric acid-producing microorganisms and an increase in various opportunistic pathogenic bacteria. The proportions of *Bacteroides* and *Firmicutes* in T2DM patients were positively correlated with blood glucose concentration (85). Many *Lactobacillus* strains play a therapeutic role in T2DM-associated periodontitis by increasing beneficial intestinal flora and reducing harmful intestinal flora. We have previously mentioned the important role of SCFAs in blood glucose regulation, and beneficial bacteria such as *Firmicutes*, *Actinobacteria, Anaerobes, Enterococcus faecalis, Lactobacillus,* and *Bifidobacteria* increase the yield of SCFAs (86). The abundance of mucus spores and SCFA-producing bacteria (*Streptococcus* and *Enterococcus faecalis*) was significantly increased following *Lactobacillus* treatment. Overall, the role of *Lactobacillus* in maintaining oral and intestinal homeostasis should not be underestimated.

## 3 Application of *Lactobacillus* on type 2 diabetic periodontitis

Despite the mechanisms described above, there are not many studies on the direct effect of *Lactobacillus* on type 2 diabetic periodontitis patients, but indirectly through the therapeutic effect of *Lactobacillus* on type 2 diabetes or periodontitis. In this study, we present the available evidence on the direct use of *Lactobacillus* in diabetic periodontitis in Table 1.

**Table 1:** Application of *Lactobacillus* on type 2 diabetes associated periodontitis.

First author (Year)	Research object	Result
Silva (87)	Rat model of diabetes and periodontitis	After *Lactobacillus* treatment, blood glucose decreased, periodontal inflammatory infiltration decreased, and bone loss decreased significantly.
Elsadek (88)	Chronic periodontitis in type-2 diabetes mellitus patients	All clinical parameters (probing depth, plaque scores, bleeding on probing, and clinical attachment level) and microbiological parameters (the assessment of detection percentage of *P. gingivalis*, *Tannerella forsythia,* and *Treponema denticola*) showed a statistically significant reduction from baseline to 3 months.
Lu (89)	Mics with diabetic periodontitis	*Lactobacillus* significantly attenuated alveolar bone loss
Malyshev (90)	Patients suffering from type 2 diabetes with moderate periodontitis	*Lactobacillus* increased local mucosal immunity

## 4 Conclusion

**Figure 1:**
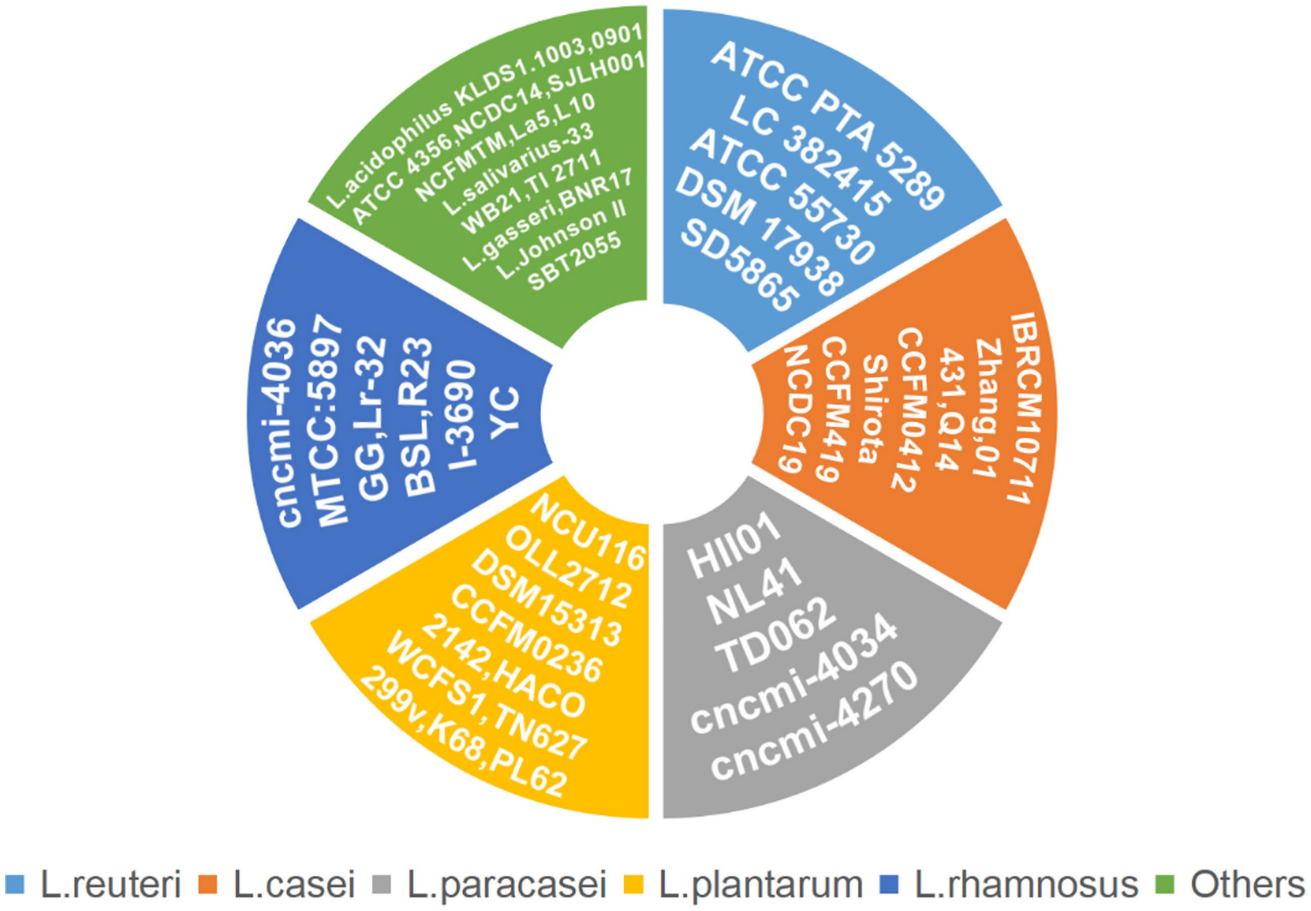
*Lactobacillus* strains associated with T2DM-associated periodontitis.

In this article, we summarized the underlying mechanism by which *Lactobacillus* is therapeutic in T2DM-associated periodontitis. *Lactobacillus* plays a direct role in treating periodontitis and indirectly treats diabetes to alleviate the symptoms of T2DM-associated periodontitis. Figure 1 lists a large number of *Lactobacillus* strains that are therapeutic for periodontitis or T2DM, while Table 2 provides more detail on the actual effectiveness of each strain.

**Table 2:** Effect of *Lactobacillus* on organism.

**Strain**	**Experiment type**	**Dose and methods**	**Application time**	**Result**
*L. reuteri* ATCC 55730	*in vivo* (clinical)	1 × 10^8^ CFU/gum by chewing	10 min/day 2w	BOP improved;GCF volume、TNF-α and IL-8 decreased
*L. reuteri* LC 382415	*in vitro*	12.5 μg/mL	1/ 3/ 6/24 h	Inhibited single- and mixed-species biofilms
*L. reuteri* DSM 17938	*in vivo* (clinical)	10^10^ CFU/d	12w	Increased ISI and DCA, improved insulin sensitivity
*L. reuteri* SD5865	*in vivo* (clinical)	2 × 10^10^ CFU/d in capsules	4w	Increased GLP-1 and GLP-2 release, higher insulin
*L. acidophilus* ATCC 4356	*in vitro*	1 × 10^5^ cells/mL (2 mL/well)	2/6/24 h	Decreased IL-1β, IL-6, and IL-8
*L. acidophilus* L10	*in vivo* (mice)	1 × 10^9^ CFU/d by oral feeding	2 w	Shortened the duration of the colonization of the oral cavity
*L. acidophilus* LA-5	*in vitro*	2.0 × 10^8^ CFU/mL	2 h	Reduced IL-1β、TNF-α and TLR4;Induced CXCL8
*L. acidophilus* NCDC14	*in vivo* (rat)	1.05 × 10^10^ CFU/d	30/60/90/120 min	Suppressed STZ-induced oxidative damage,decreased NO
*L. acidophilus* SJLH001	*in vitro*	10^9^ CFU/mouse/d	20w	Improved glucose homeostasis
*L. acidophilus* KLDS1.1003; KLDS1.0901	*in vivo* (mice)	1× 10^9^ CFU/d	6w	Lowered inflammation cytokines,downregulated the expression of (GSK-3β, FAS and SREBP-1c)
*L. acidophilus* NCFM	*in vivo* (clinical)	Not mentioned	4w	Preserved insulin sensitivity
*L. plantarum* K68	*in vivo* (rat)	1 × 10^9^ CFU/0.5 mL/d	8w	IL-1、IL-6 and TNF-α were controlled
*L. plantarum* NCU116	*in vivo* (rat)	10^9^ 10^10^ CFU/kg	5w	Restored liver function and oxidative stress
*L. plantarum* OLL2712	*in vitro*	Not mentioned	8、14、16 h	Decreased IL-10、IL-12
*L. plantarum* CCFM0236	*in vivo* (mice)	8 × 10^9^ CFU/mL 0.25 mL/d	5w	Increased the activities of glutathione peroxidase
*L. plantarum* 2,142	*in vitro*	Not mentioned	Not mentioned	Decreased IL-8、TNF-α
*L. plantarum* DSM 15313	*in vivo* (mice)	7 × 10^9^ CFU/d by feeding	20w	Lowered fasting plasma glucose levels
*L. plantarum* TN627	*in vivo* (rat)	0.9 × 10^9^ CFU/mL 2 mL	10/20/30/60/90 min	Reduce the activities and level of plasma glucose
*L. plantarum* PL62	*in vivo* (mice)	10^7^ or 10^9^ CFU/d	8w	Reduced the blood glucose and body weights
*L. plantarum* 299v	*in vivo* (clinical)	2 × 10^10^ CFU/d orally	6w	Decreased *F* (2)-isoprostanes and IL-6
*L. plantarum* WCFS1	*in vivo* (clinical)	10^12^ CFU by injecting	6 h	Attenuated the increase in epithelial permeability
*L. casei strain* Shirota	*in vivo* (mice)	10 μg/mL	24 h	TNF-α, IL-12, IL-10, and IL-6 decreased
	*in vivo* (mice)	0.05% (w/w)	5w	Suppressed the elevation of plasma LBP levels
	*in vivo* (rat)	1 × 10^9^ CFU by injecting	30/60/90/120 min	Lowered blood sugar
*L. casei* CCFM419	*in vivo* (mice)	10^8^, 10^9^, and 10^10^ CFU	4w	ImprovedFBG, postprandial blood glucose, glucose intolerance, and insulin resistance.
*L. casei* 01	*in vivo* (clinical)	10^8^ CFU/day capsules	8w	Decreased fetuin-A level, insulin concentration, and insulin resistance
*L. casei* CRL 431	*in vivo* (mice)	8 ± 2 × 10^8^ CFU/mL 3–4 mL of milk /d	60d	Improved the biochemical and immunologic parameters altered
*L. case* Q14	*in vivo* (rat)	2.3 × 10^9^ CFU/mL 0.5 mL/kg	6w	Improved blood glucose, reduced gluconeogenesis
*L. casei* NCDC19	*in vivo* (rat)	1.05 × 10^10^ CFU/d	30/60/90/120 min	Suppressed STZ-induced oxidative damage, decreased NO
*L. casei* CCFM0412	*in vivo* (clinical)	10^9^ CFU/d by injecting	12w	Reduced postprandial blood glucose
*L. casei* Zhang	*in vivo* (rat)	1 × 10^9^ CFU/d orally	16 h	The level of LPS、iNOS decreased
*L. casei* IBRC_M10711	*in vitro*	10^8^ CFU/mL	4 h	Inhibited IDE activity
*L. rhamnosus* I-3690	*in vivo* (mice)	10^8^ cells/d	12w	Improved glucose insulin homeostasis
*L. rhamnosus* YC	*in vivo* (mice)	1 × 10^9^ CFU/d 6d/w	16w	Reduced the fasting and postprandial blood sugar levels, improved glucose tolerance
*L. rhamnosus* GG	*in vivo* (rat)	2z lyophilized GG cells	9w	Lowered HbA1C and improved glucose tolerance
	*in vivo* (rat)	diet with 0.5% viable GG cells	6w	Inhibited fasting and postprandial blood glucose
	vivo (clinical)	2×10^9^ cells	4w	Increased MMP-9 and decreased TIMP-1 levels
	*in vivo* (mouse)	1 × 10^8^ CFU	4w	Improved glucose tolerance
*L. rhamnosus* BSL	*in vivo* (rat)	10^9^ CFU/d	30d	Reduced G6pc, manage blood glucose level
*L. rhamnosus* MTCC:5957	*in vivo* (rat)	10^9^ CFU/mL milk by feeding	6w	Improved glucose metabolism, serum inflammation status, oxidative stress
*L. rhamnosus* CNCM I-4036	*in vivo* (rat)	10^10^ CFU orally	30 d	Increased the ratio P-Akt/Akt and NF-kB protein levels.
*L. rhamnosus* Lr-3	*in vitro*	2.0 × 10^8^ CFU/mL	2 h	Reduced IL-1β、TNF-α and TLR4;Induced CXCL8
*L. paracasei* CNCM I-4034	*in vivo* (rat)	10^10^ CFU orally	30 d	Increased the ratio P-Akt/Akt and NF-kB protein levels.
*L. paracasei* CNCM I-4270	*in vivo* (mice)	10^8^ cells/d	12w	Improved glucose insulin homeostasis
*L. gasseri* SBT2055	*in vivo* (mice)	5 × 10^8^ CFU/g	24w	Inhibited lipogenic gene upregulation
*L. gasseri* BNR17	*in vivo* (mice)	10^9/10^CFU twice a day	10w	Upregulate the expression of GLUT4
*L. gasseri* ATCC 33323	*in vivo* (mice)	1 × 10^9^ CFU/2d by gavaging	8w	Attenuated weight gain and improve glucose-insulin homeostasis
*L. johnsonii* NCC 533				
*L. salivarius* 33	*in vivo* (clinical)	a capsule/day	12w	Ratios of Bacteroides-Prevotella-Porphyromonas group increased
*L. salivarius* WB21	*in vivo* (clinical)	2.01 × 10^9^ CFU/d in tablets	8 w	Five selected periodontopathic bacteria was decreased
*L. salivarius* TI 2711	*in vivo* (clinical)	2 × 10^7^ CFU/d in tablets	4 /8 w	Black-pigmented anaerobic rods decreased

**Figure 2:**
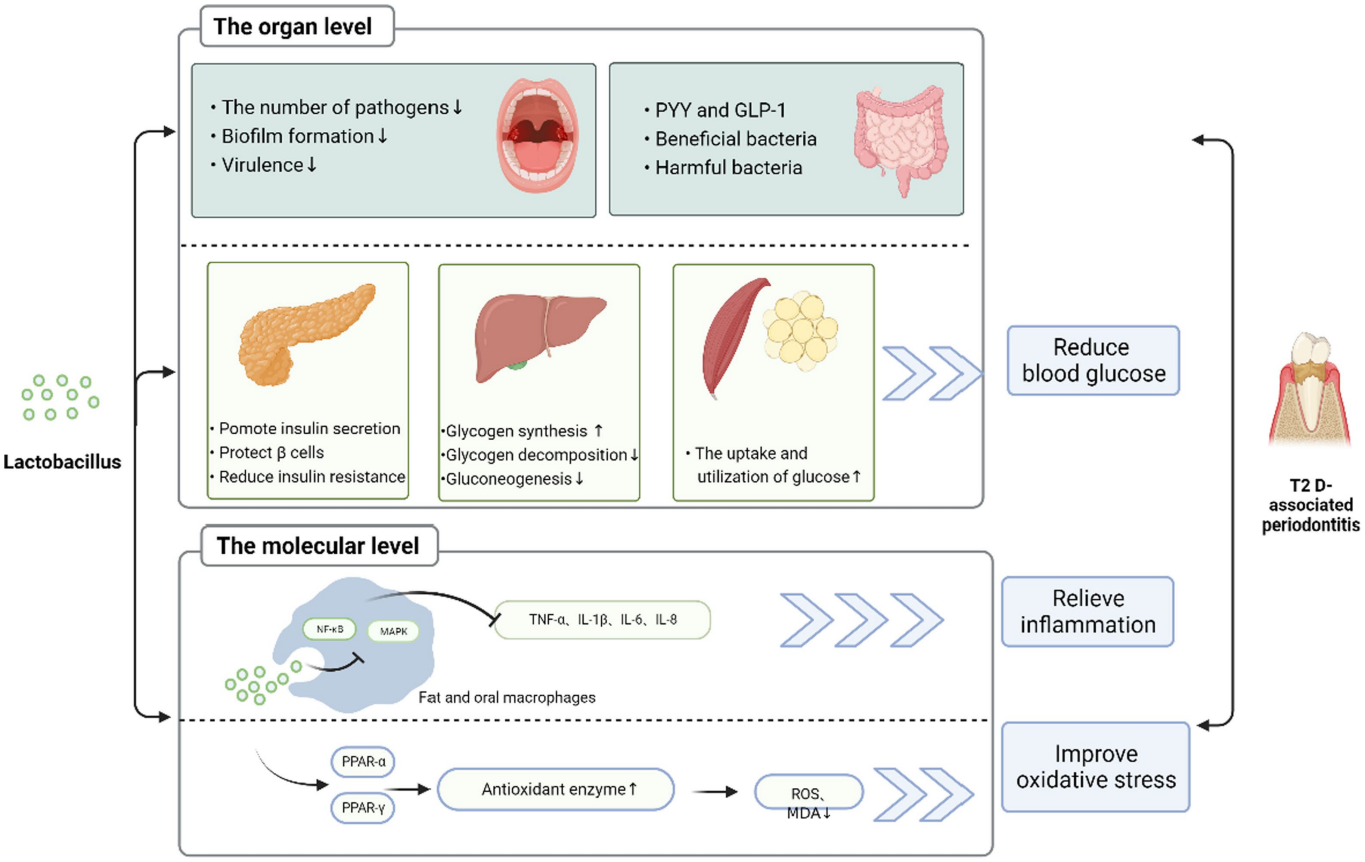
Possible mechanism of *Lactobacillus* improving diabetes mellitus with T2DM-associated periodontitis *Lactobacillus* can improve T2DM-associated periodontitis in various ways. (1) At the tissue level, *Lactobacillus* can promote insulin secretion and protect the pancreas *β* cells, reduce insulin resistance in the pancreas; increase glycogen synthesis; reduce glycogen decomposition and gluconeogenesis in the liver; increase PYY and GLP-1; increase beneficial bacteria and reduce harmful bacteria in the gut; increase glucose utilization in fat and muscle groups. Through these ways, it can reduce blood sugar, thereby reducing inflammation. In addition, *Lactobacillus* reduces the number of pathogenic bacteria, biofilm formation, and virulence in oral cavity and improves T2DM-associated periodontitis. (2) At the molecular level, *Lactobacillus* through MAPK and NF-κB signaling pathway, reduces the level of inflammatory factors. It is also possible to adjust PPAR-α and PPAR-γ to upregulate the level of antioxidant enzymes, thereby reducing ROS and MDA, improving oxidative stress, and ameliorating periodontitis in type II diabetes.

*Lactobacillus* improves the activity of periodontitis associated with T2DM. As shown in Figure 2, this may be related to intricate and complex mechanisms, including but not limited to reducing inflammation, modulating oxidative stress, improving glucose metabolism, direct antagonism of pathogenic bacteria, and modulating the microbiota. *Lactobacillus* may reduce inflammation by downregulating virulence gene expression, modulating cytokine levels, inhibiting NO production, reducing intestinal permeability, and enhancing intestinal barrier function. In terms of improving glucose metabolism, *Lactobacillus* can directly inhibit glucose synthesis, reduce glucose production, decrease insulin resistance, and protect blood glucose beta cells. It has also been suggested that lactobacilli can exert glucose-lowering effects by reducing bacterial translocation and modulating the intestinal ACSL3 pathway (91), but the relevant evidence is not sufficient. Most importantly, *Lactobacillus* intervention significantly altered the composition of the microbial community by reducing the number of pathogenic bacteria associated with T2DM-associated periodontitis in the oral microbiota and gut microbiota and increasing the number of beneficial bacteria, thereby showing therapeutic activity against T2DM-associated periodontitis. In summary, *Lactobacillus* are a promising option in the treatment of T2DM-associated periodontitis because they play a key role in host metabolism, regulate intestinal microecology, and reduce inflammation. Clinicians may consider including *Lactobacillus* in their treatment plans when treating T2DM-associated periodontitis.

Nevertheless, some studies contradict our conclusions. A previous study found that eating soybean milk containing *L. plantarum* had no effect on serum Apn or inflammation (92). Similar results were reported by Hatakka et al. (93) in their study, probiotic supplementation did not result in significant changes in cytokines (such as TNF-a) within or between groups. This may be due to the beneficial effects of *Lactobacillus* supplementation, which may be related to the following factors: the specific *Lactobacillus* strains, the formulation of *Lactobacillus* probiotics (single or multiple strains, or with no prebiotics), the intervention time, dosage, and other factors. The anti-inflammatory properties of *Lactobacillus* are strain- and time-specific. Many studies have recommended regular consumption of preparations containing *Lactobacillus* to maintain health benefits. However, there are many strains of *Lactobacillus*, and the optimal dosage and frequency of each strain have not been explored, which may be the reason for the limited use of *Lactobacillus* in the clinic. Hence, further studies are needed to evaluate the best combination and application of *Lactobacillus* strains or synthetic probiotics containing *Lactobacillus* to prolong the effect of *Lactobacillus* on T2DM-associated periodontitis individuals and to explore the deeper mechanisms of T2DM-associated periodontitis treatment. In addition, there is not much evidence on the direct treatment of T2DM-associated periodontitis by *Lactobacillus*, but more studies on the significant effects on T2DM and the reduction in the effect of AGEs in T2DM-associated periodontitis. In addition, most of the current studies are based on animals, and there is a lack of research on patients with T2DM-associated periodontitis, so more research on this aspect is needed in future.

## Author contributions

SC was responsible for literature review, data sorting, and draft writing. YZ was responsible for determining the title, revising, and approving the manuscript. All authors contributed to the article and approved the submitted version.

## References

[1] Zhou X Zhang W Liu X Zhang W Li Y. Interrelationship between diabetes and periodontitis: role of hyperlipidemia. Arch Oral Biol. (2015) 60:667–74. doi: 10.1016/j.archoralbio.2014.11.008, PMID: 25443979

[2] Papapanou PN Sanz M Buduneli N Dietrich T Feres M Fine DH. Periodontitis: consensus report of workgroup 2 of the 2017 world workshop on the classification of periodontal and Peri-implant diseases and conditions. J Periodontol. (2018) 89 Suppl 1:S173–82. doi: 10.1002/JPER.17-0721, PMID: 29926951

[3] Ide R Hoshuyama T Wilson D Takahashi K Higashi T. Periodontal disease and incident diabetes: a seven-year study. J Dent Res. (2011) 90:41–6. doi: 10.1177/0022034510381902 21041549

[4] Graziani F Gennai S Solini A Petrini M. A systematic review and meta-analysis of epidemiologic observational evidence on the effect of periodontitis on diabetes an update of the EFP-AAP review. J Clin Periodontol. (2018) 45:167–87. doi: 10.1111/jcpe.12837, PMID: 29277926

[5] Polepalle T Moogala S Boggarapu S Pesala DS Palagi FB. Acute phase proteins and their role in periodontitis: a review. J Clin Diagn Res. (2015) 9:ZE01–5. doi: 10.7860/JCDR/2015/15692.6728, PMID: 26674303 PMC4668538

[6] Steward FC Mott RL. Cells, solutes, and growth: salt accumulation in plants reexamined. Int Rev Cytol. (1970) 28:275–370. doi: 10.1016/s0074-7696(08)62546-2 4244668

[7] Lamont RJ Koo H Hajishengallis G. The oral microbiota: dynamic communities and host interactions. Nat Rev Microbiol. (2018) 16:745–59. doi: 10.1038/s41579-018-0089-x, PMID: 30301974 PMC6278837

[8] Iatcu CO Steen A Covasa M. Gut microbiota and complications of Type-2 diabetes. Nutrients. (2021) 14:166. doi: 10.3390/nu14010166 35011044 PMC8747253

[9] Sookkhee S Chulasiri M Prachyabrued W. Lactic acid bacteria from healthy oral cavity of Thai volunteers: inhibition of oral pathogens. J Appl Microbiol. (2001) 90:172–9. doi: 10.1046/j.1365-2672.2001.01229.x, PMID: 11168719

[10] Qin J Li Y Cai Z Li S Zhu J Zhang F. A metagenome-wide association study of gut microbiota in type 2 diabetes. Nature. (2012) 490:55–60. doi: 10.1038/nature11450, PMID: 23023125

[11] Hill C Guarner F Reid G Gibson GR Merenstein DJ Pot B. Expert consensus document. The international scientific Association for Probiotics and Prebiotics consensus statement on the scope and appropriate use of the term probiotic. Nat Rev Gastroenterol Hepatol. (2014) 11:506–14. doi: 10.1038/nrgastro.2014.66, PMID: 24912386

[12] Kim S Woo GJ. Prevalence and characterization of antimicrobial-resistant *Escherichia coli* isolated from conventional and organic vegetables. Foodborne Pathog Dis. (2014) 11:815–21. doi: 10.1089/fpd.2014.1771, PMID: 25140978

[13] Kandler O. Archaebacteria and phylogeny of organisms. Naturwissenschaften. (1981) 68:183–92. doi: 10.1007/BF01047198, PMID: 6168919

[14] Colloca ME Ahumada MC Lopez ME Nader-Macias ME. Surface properties of lactobacilli isolated from healthy subjects. Oral Dis. (2000) 6:227–33. doi: 10.1111/j.1601-0825.2000.tb00118.x, PMID: 10918560

[15] Zhao X Zhong X Liu X Wang X Gao X. Therapeutic and improving function of lactobacilli in the prevention and treatment of cardiovascular-related diseases: a novel perspective from gut microbiota. Front Nutr. (2021) 8:693412. doi: 10.3389/fnut.2021.693412, PMID: 34164427 PMC8215129

[16] Marcotte H Larsson PG Andersen KK Zuo F Mikkelsen LS Brandsborg E. An exploratory pilot study evaluating the supplementation of standard antibiotic therapy with probiotic lactobacilli in south African women with bacterial vaginosis. BMC Infect Dis. (2019) 19:824. doi: 10.1186/s12879-019-4425-1, PMID: 31533663 PMC6751625

[17] Nami Y Haghshenas B Haghshenas M Abdullah N Yari KA. The prophylactic effect of probiotic Enterococcus lactis IW5 against different human Cancer cells. Front Microbiol. (2015) 6:1317. doi: 10.3389/fmicb.2015.01317, PMID: 26635778 PMC4659899

[18] Ho HE Bunyavanich S. Microbial adjuncts for food allergen immunotherapy. Curr Allergy Asthma Rep. (2019) 19:25. doi: 10.1007/s11882-019-0859-1, PMID: 30903301

[19] Gionchetti P Rizzello F Venturi A Brigidi P Matteuzzi D Bazzocchi G. Oral bacteriotherapy as maintenance treatment in patients with chronic pouchitis: a double-blind, placebo-controlled trial. Gastroenterology. (2000) 119:305–9. doi: 10.1053/gast.2000.9370, PMID: 10930365

[20] Madsen K Cornish A Soper P McKaigney C Jijon H Yachimec C. Probiotic bacteria enhance murine and human intestinal epithelial barrier function. Gastroenterology. (2001) 121:580–91. doi: 10.1053/gast.2001.27224 11522742

[21] Loskutova IE. Effectiveness of using Maliutka and Malysh adapted propionic-acidophilus mixtures in the combined treatment of congenital hypotrophy. Vopr Pitan. (1985) 3:17–20. 4036072

[22] Gao X Jiang S Koh D Hsu CY. Salivary biomarkers for dental caries. Periodontol. (2000) 70:128–41. doi: 10.1111/prd.12100 26662487

[23] Nguyen T Brody H Radaic A Kapila Y. Probiotics for periodontal health-current molecular findings. Periodontol. (2000) 2021:254–67. doi: 10.1111/prd.12382 PMC8448672 34463979

[24] Luoto R Laitinen K Nermes M Isolauri E. Impact of maternal probiotic-supplemented dietary counselling on pregnancy outcome and prenatal and postnatal growth: a double-blind, placebo-controlled study. Br J Nutr. (2010) 103:1792–9. doi: 10.1017/S0007114509993898, PMID: 20128938

[25] Moroti C Souza Magri LF de Rezende CM Cavallini DC Sivieri K. Effect of the consumption of a new symbiotic shake on glycemia and cholesterol levels in elderly people with type 2 diabetes mellitus. Lipids Health Dis. (2012) 11:29. doi: 10.1186/1476-511X-11-29, PMID: 22356933 PMC3305430

[26] Jebin AA Nisha KJ Padmanabhan S. Oral microbial shift following 1-month supplementation of probiotic chewable tablets containing *Lactobacillus reuteri* UBLRu-87 as an adjunct to phase 1 periodontal therapy in chronic periodontitis patients: a randomized controlled clinical trial. Contemp Clin Dent. (2021) 12:121–7. doi: 10.4103/ccd.ccd_135_20, PMID: 34220150 PMC8237819

[27] Albuquerque-Souza E Balzarini D Ando-Suguimoto ES Ishikawa KH Simionato MRL Holzhausen M. Probiotics alter the immune response of gingival epithelial cells challenged by *Porphyromonas gingivalis*. J Periodontal Res. (2019) 54:115–27. doi: 10.1111/jre.12608, PMID: 30284741

[28] Sturm A Baumgart DC d'Heureuse JH Hotz A Wiedenmann B Dignass AU. CXCL8 modulates human intestinal epithelial cells through a CXCR1 dependent pathway. Cytokine. (2005) 29:42–8. doi: 10.1016/j.cyto.2004.09.007, PMID: 15579377

[29] Albuquerque-Souza E Ishikawa KH Amado PP Nicoli JR Holzhausen M Mayer MPA. Probiotics improve re-epithelialization of scratches infected by *Porphyromonas gingivalis* through up-regulating CXCL8-CXCR1/CXCR2 axis. Anaerobe. (2021) 72:102458. doi: 10.1016/j.anaerobe.2021.102458, PMID: 34547426

[30] Stamatova I Meurman JH. Probiotics and periodontal disease. Periodontology. (2000) 51:141–51. doi: 10.1111/j.1600-0757.2009.00305.x 19878473

[31] Saglam M Kantarci A Dundar N Hakki SS. Clinical and biochemical effects of diode laser as an adjunct to nonsurgical treatment of chronic periodontitis: a randomized, controlled clinical trial. Lasers Med Sci. (2014) 29:37–46. doi: 10.1007/s10103-012-1230-0, PMID: 23161345

[32] Reynolds JJ. Collagenases and tissue inhibitors of metalloproteinases: a functional balance in tissue degradation. Oral Dis. (1996) 2:70–6. doi: 10.1111/j.1601-0825.1996.tb00206.x 8957940

[33] Tüter G Serdar M Kurtiş B Walker SG Atak A Toyman U. Effects of scaling and root planing and subantimicrobial dose doxycycline on gingival crevicular fluid levels of matrix metalloproteinase-8, −13 and serum levels of HsCRP in patients with chronic periodontitis. J Periodontol. (2010) 81:1132–9. doi: 10.1902/jop.2010.090694, PMID: 20370419

[34] Ince G Gursoy H Ipci SD Cakar G Emekli-Alturfan E Yilmaz S. Clinical and biochemical evaluation of lozenges containing *Lactobacillus reuteri* as an adjunct to non-surgical periodontal therapy in chronic periodontitis. J Periodontol. (2015) 86:746–54. doi: 10.1902/jop.2015.140612 25741580

[35] Cloitre A Halgand B Sourice S Caillon J Huck O Bugueno IM. IL-36gamma is a pivotal inflammatory player in periodontitis-associated bone loss. Sci Rep. (2019) 9:19257. doi: 10.1038/s41598-019-55595-9, PMID: 31848404 PMC6917751

[36] Sulijaya B Takahashi N Yamazaki K. Lactobacillus-derived bioactive metabolites for the regulation of periodontal health: evidences to clinical setting. Molecules. (2020) 25:2088. doi: 10.3390/molecules25092088 32365716 PMC7248875

[37] Huang HY Korivi M Tsai CH Yang JH Tsai YC. Supplementation of *Lactobacillus plantarum* K68 and fruit-vegetable ferment along with high fat-fructose diet attenuates metabolic syndrome in rats with insulin resistance. Evid Based Complement Alternat Med. (2013) 2013:943020. doi: 10.1155/2013/943020, PMID: 23690866 PMC3652198

[38] Wang G Li X Zhao J Zhang H Chen W. *Lactobacillus casei* CCFM419 attenuates type 2 diabetes via a gut microbiota dependent mechanism. Food Funct. (2017) 8:3155–64. doi: 10.1039/c7fo00593h, PMID: 28782784

[39] Pena JA Versalovic J. *Lactobacillus rhamnosus* GG decreases TNF-alpha production in lipopolysaccharide-activated murine macrophages by a contact-independent mechanism. Cell Microbiol. (2003) 5:277–85. doi: 10.1046/j.1462-5822.2003.t01-1-00275.x, PMID: 12675685

[40] Kazmierczyk-Winciorek M Nedzi-Gora M Slotwinska SM. The immunomodulating role of probiotics in the prevention and treatment of oral diseases. Cent Eur J Immunol. (2021) 46:99–104. doi: 10.5114/ceji.2021.104412, PMID: 33897290 PMC8056348

[41] de Kort S Keszthelyi D Masclee AA. Leaky gut and diabetes mellitus: what is the link? Obes Rev. (2011) 12:449–58. doi: 10.1111/j.1467-789X.2010.00845.x, PMID: 21382153

[42] van Baarlen P Troost FJ van Hemert S van der Meer C de Vos WM de Groot PJ. Differential NF-kappaB pathways induction by *Lactobacillus plantarum* in the duodenum of healthy humans correlating with immune tolerance. Proc Natl Acad Sci U S A. (2009) 106:2371–6. doi: 10.1073/pnas.0809919106, PMID: 19190178 PMC2650163

[43] Karczewski J Troost FJ Konings I Dekker J Kleerebezem M Brummer RJ. Regulation of human epithelial tight junction proteins by *Lactobacillus plantarum* in vivo and protective effects on the epithelial barrier. Am J Physiol Gastrointest Liver Physiol. (2010) 298:G851–9. doi: 10.1152/ajpgi.00327.2009, PMID: 20224007

[44] Srutkova D Schwarzer M Hudcovic T Zakostelska Z Drab V Spanova A. *Bifidobacterium longum* CCM 7952 promotes epithelial barrier function and prevents acute DSS-induced colitis in strictly strain-specific manner. PLoS One. (2015) 10:e0134050. doi: 10.1371/journal.pone.0134050, PMID: 26218526 PMC4517903

[45] Seminario-Amez M Lopez-Lopez J Estrugo-Devesa A Ayuso-Montero R Jane-Salas E. Probiotics and oral health: a systematic review. Med Oral Patol Oral Cir Bucal. (2017) 22:e282–8. doi: 10.4317/medoral.21494, PMID: 28390121 PMC5432076

[46] Widyarman AS Theodorea CF. Novel indigenous probiotic *Lactobacillus reuteri* strain produces anti-biofilm Reuterin against pathogenic periodontal Bacteria. Eur J Dent. (2022) 16:96–101. doi: 10.1055/s-0041-1731591, PMID: 34303315 PMC8890917

[47] Wilson RM Walker JM Yin K. Different concentrations of *Lactobacillus acidophilus* cell free filtrate have differing anti-biofilm and immunomodulatory effects. Front Cell Infect Microbiol. (2021) 11:737392. doi: 10.3389/fcimb.2021.737392, PMID: 34589444 PMC8473619

[48] Sczepanik FSC Grossi ML Casati M Goldberg M Glogauer M Fine N. Periodontitis is an inflammatory disease of oxidative stress: we should treat it that way. Periodontol. (2000) 84:45–68. doi: 10.1111/prd.12342, PMID: 32844417

[49] Chang YC Chuang LM. The role of oxidative stress in the pathogenesis of type 2 diabetes: from molecular mechanism to clinical implication. Am J Transl Res. (2010) 2:316–31. doi: 10.4168/aair.2010.2.3.183 PMID: 20589170 PMC2892404

[50] Lin MY Chang FJ. Antioxidative effect of intestinal bacteria *Bifidobacterium longum* ATCC 15708 and *Lactobacillus acidophilus* ATCC 4356. Digest Dis Sci. (2000) 45:1617–22. doi: 10.1023/A:1005577330695 11007114

[51] Yokoji-Takeuchi M Takahashi N Yamada-Hara M Sulijaya B Tsuzuno T Aoki-Nonaka Y. A bacterial metabolite induces Nrf2-mediated anti-oxidative responses in gingival epithelial cells by activating the MAPK signaling pathway. Arch Oral Biol. (2020) 110:104602. doi: 10.1016/j.archoralbio.2019.104602, PMID: 31734544

[52] Dang F Jiang Y Pan R Zhou Y Wu S Wang R. Administration of *Lactobacillus paracasei* ameliorates type 2 diabetes in mice. Food Funct. (2018) 9:3630–9. doi: 10.1039/c8fo00081f, PMID: 29961787

[53] Zeng Z Yuan Q Yu R Zhang J Ma H Chen S. Ameliorative effects of probiotic *Lactobacillus paracasei* NL41 on insulin sensitivity, oxidative stress, and Beta-cell function in a type 2 diabetes mellitus rat model. Mol Nutr Food Res. (2019) 63:e1900457. doi: 10.1002/mnfr.201900457, PMID: 31433912

[54] Herrera BS Martins-Porto R Maia-Dantas A Campi P Spolidorio LC Costa SK. iNOS-derived nitric oxide stimulates osteoclast activity and alveolar bone loss in ligature-induced periodontitis in rats. J Periodontol. (2011) 82:1608–15. doi: 10.1902/jop.2011.100768, PMID: 21417589 PMC3361509

[55] Han CY. Roles of reactive oxygen species on insulin resistance in adipose tissue. Diabetes Metab J. (2016) 40:272–9. doi: 10.4093/dmj.2016.40.4.272, PMID: 27352152 PMC4995181

[56] Liu WC Yang MC Wu YY Chen PH Hsu CM Chen LW. *Lactobacillus plantarum* reverse diabetes-induced Fmo3 and ICAM expression in mice through enteric dysbiosis-related c-Jun NH2-terminal kinase pathways. PLoS One. (2018) 13:e0196511. doi: 10.1371/journal.pone.0196511, PMID: 29851956 PMC5978885

[57] Zhang Y Guo X Guo J He Q Li H Song Y. *Lactobacillus casei* reduces susceptibility to type 2 diabetes via microbiota-mediated body chloride ion influx. Sci Rep. (2014) 4:5654. doi: 10.1038/srep05654, PMID: 25133590 PMC4135721

[58] Harisa IG Taha IE Khalil FA Salem MM. Oral Administration of *Lactobacillus Acidophilus* Restores Nitric Oxide Level in diabetic rats. Austral J Basic Applied Sci. (2009)

[59] Yao K Zeng L He Q Wang W Lei J Zou X. Effect of probiotics on glucose and lipid metabolism in type 2 diabetes mellitus: a Meta-analysis of 12 randomized controlled trials. Med Sci Monit. (2017) 23:3044–53. doi: 10.12659/msm.902600, PMID: 28638006 PMC5491138

[60] Yan F Li N Shi J Li H Yue Y Jiao W. *Lactobacillus acidophilus* alleviates type 2 diabetes by regulating hepatic glucose, lipid metabolism and gut microbiota in mice. Food Funct. (2019) 10:5804–15. doi: 10.1039/c9fo01062a, PMID: 31461095

[61] Farida E Nuraida L Giriwono PE Jenie BSL. *Lactobacillus rhamnosus* reduces blood glucose level through downregulation of gluconeogenesis gene expression in Streptozotocin-induced diabetic rats. Int J Food Sci. (2020) 2020:6108575. doi: 10.1155/2020/6108575, PMID: 32399477 PMC7201496

[62] Yadav R Dey DK Vij R Meena S Kapila R Kapila S. Evaluation of anti-diabetic attributes of *Lactobacillus rhamnosus* MTCC: 5957, *Lactobacillus rhamnosus* MTCC: 5897 and *Lactobacillus fermentum* MTCC: 5898 in streptozotocin induced diabetic rats. Microb Pathog. (2018) 125:454–62. doi: 10.1016/j.micpath.2018.10.015, PMID: 30316007

[63] Zhang L Zhou W Zhan L Hou S Zhao C Bi T. Fecal microbiota transplantation alters the susceptibility of obese rats to type 2 diabetes mellitus. Aging (Albany NY). (2020) 12:17480–502. doi: 10.18632/aging.103756, PMID: 32920548 PMC7521520

[64] Li X Wang E Yin B Fang D Chen P Wang G. Effects of *Lactobacillus casei* CCFM419 on insulin resistance and gut microbiota in type 2 diabetic mice. Benef Microbes. (2017) 8:421–32. doi: 10.3920/BM2016.0167, PMID: 28504567

[65] Tabuchi M Ozaki M Tamura A Yamada N Ishida T Hosoda M. Antidiabetic effect of Lactobacillus GG in streptozotocin-induced diabetic rats. Biosci Biotechnol Biochem. (2003) 67:1421–4. doi: 10.1271/bbb.67.1421 12843677

[66] Honda K Moto M Uchida N He F Hashizume N. Anti-diabetic effects of lactic acid bacteria in normal and type 2 diabetic mice. J Clin Biochem Nutr. (2012) 51:96–101. doi: 10.3164/jcbn.11-07, PMID: 22962525 PMC3432833

[67] Lihn AS Pedersen SB Richelsen B. Adiponectin: action, regulation and association to insulin sensitivity. Obes Rev. (2005) 6:13–21. doi: 10.1111/j.1467-789X.2005.00159.x 15655035

[68] Kim SW Park KY Kim B Kim E Hyun CK. *Lactobacillus rhamnosus* GG improves insulin sensitivity and reduces adiposity in high-fat diet-fed mice through enhancement of adiponectin production. Biochem Biophys Res Commun. (2013) 431:258–63. doi: 10.1016/j.bbrc.2012.12.121, PMID: 23313485

[69] Newsholme P Cruzat VF Keane KN Carlessi R de Bittencourt PI Jr. Molecular mechanisms of ROS production and oxidative stress in diabetes. Biochem J. (2016) 473:4527–50. doi: 10.1042/BCJ20160503C 27941030

[70] Matsuzaki T Yamazaki R Hashimoto S Yokokura T. Antidiabetic effects of an oral administration of *Lactobacillus casei* in a non-insulin-dependent diabetes mellitus (NIDDM) model using KK-ay mice. Endocr J. (1997) 44:357–65. doi: 10.1507/endocrj.44.357, PMID: 9279510

[71] Cani PD Lecourt E Dewulf EM Sohet FM Pachikian BD Naslain D. Gut microbiota fermentation of prebiotics increases satietogenic and incretin gut peptide production with consequences for appetite sensation and glucose response after a meal. Am J Clin Nutr. (2009) 90:1236–43. doi: 10.3945/ajcn.2009.28095, PMID: 19776140

[72] Simon MC Strassburger K Nowotny B Kolb H Nowotny P Burkart V. Intake of *Lactobacillus reuteri* improves incretin and insulin secretion in glucose-tolerant humans: a proof of concept. Diabetes Care. (2015) 38:1827–34. doi: 10.2337/dc14-2690, PMID: 26084343

[73] Yadav H Jain S Sinha PR. Oral administration of dahi containing probiotic Lactobacillus acidophilus and *Lactobacillus casei* delayed the progression of streptozotocin-induced diabetes in rats. J Dairy Res. (2008) 75:189–95. doi: 10.1017/S0022029908003129, PMID: 18474136

[74] Puddu A Sanguineti R Montecucco F Viviani GL. Evidence for the gut microbiota short-chain fatty acids as key pathophysiological molecules improving diabetes. Mediat Inflamm. (2014) 2014:162021. doi: 10.1155/2014/162021, PMID: 25214711 PMC4151858

[75] Hintao J Teanpaisan R Chongsuvivatwong V Ratarasan C Dahlen G. The microbiological profiles of saliva, supragingival and subgingival plaque and dental caries in adults with and without type 2 diabetes mellitus. Oral Microbiol Immunol. (2007) 22:175–81. doi: 10.1111/j.1399-302X.2007.00341.x, PMID: 17488443

[76] Koll-Klais P Mandar R Leibur E Marcotte H Hammarstrom L Mikelsaar M. Oral lactobacilli in chronic periodontitis and periodontal health: species composition and antimicrobial activity. Oral Microbiol Immunol. (2005) 20:354–61. doi: 10.1111/j.1399-302X.2005.00239.x, PMID: 16238595

[77] Imlay JA. The molecular mechanisms and physiological consequences of oxidative stress: lessons from a model bacterium. Nat Rev Microbiol. (2013) 11:443–54. doi: 10.1038/nrmicro3032, PMID: 23712352 PMC4018742

[78] Cornacchione LP Klein BA Duncan MJ Hu LT. Interspecies inhibition of *Porphyromonas gingivalis* by yogurt-derived *Lactobacillus delbrueckii* requires active pyruvate oxidase. Appl Environ Microbiol. (2019) 85:e01271–19. doi: 10.1128/AEM.01271-19 31285191 PMC6715850

[79] Ding Q Sun X Cao S Zhao C Wang Y Wang X. Heat-killed *Lactobacillus acidophilus* mediates *Fusobacterium nucleatum* induced pro-inflammatory responses in epithelial cells. FEMS Microbiol Lett. (2021) 368:160. doi: 10.1093/femsle/fnaa160 33693760

[80] Suzuki N Tanabe K Takeshita T Yoneda M Iwamoto T Oshiro S. Effects of oil drops containing *Lactobacillus salivarius* WB21 on periodontal health and oral microbiota producing volatile sulfur compounds. J Breath Res. (2012) 6:017106. doi: 10.1088/1752-7155/6/1/017106, PMID: 22368259

[81] Mayanagi G Kimura M Nakaya S Hirata H Sakamoto M Benno Y. Probiotic effects of orally administered *Lactobacillus salivarius* WB21-containing tablets on periodontopathic bacteria: a double-blinded, placebo-controlled, randomized clinical trial. J Clin Periodontol. (2009) 36:506–13. doi: 10.1111/j.1600-051X.2009.01392.x, PMID: 19453574

[82] Jabri B Iken M Ait-Ou-Amar S Rida S Bouziane A Ennibi OK. Candida albicans and Candida dubliniensis in periodontitis in adolescents and young adults. Int J Microbiol. (2022) 2022:4625368–8. doi: 10.1155/2022/4625368, PMID: 35058983 PMC8766183

[83] Payne S Gibson G Wynne A Hudspith B Brostoff J Tuohy K. *In vitro* studies on colonization resistance of the human gut microbiota to Candida albicans and the effects of tetracycline and *Lactobacillus plantarum* LPK. Curr Issues Intest Microbiol. (2003) 4:1–8. PMID: 12691257

[84] Koliarakis I Messaritakis I Nikolouzakis TK Hamilos G Souglakos J Tsiaoussis J. Oral Bacteria and intestinal Dysbiosis in colorectal Cancer. Int J Mol Sci. (2019) 20:4146. doi: 10.3390/ijms20174146 31450675 PMC6747549

[85] Larsen N Vogensen FK van den Berg FW Nielsen DS Andreasen AS Pedersen BK. Gut microbiota in human adults with type 2 diabetes differs from non-diabetic adults. PLoS One. (2010) 5:e9085. doi: 10.1371/journal.pone.0009085, PMID: 20140211 PMC2816710

[86] Gao H Wen JJ Hu JL Nie QX Chen HH Xiong T. Polysaccharide from fermented *Momordica charantia* L. with *Lactobacillus plantarum* NCU116 ameliorates type 2 diabetes in rats. Carbohydr Polym. (2018) 201:624–33. doi: 10.1016/j.carbpol.2018.08.075, PMID: 30241862

[87] Silva DNA Cruz NTS Martins AA Silva RCM Almeida HC Costa HES. Probiotic *Lactobacillus rhamnosus* EM1107 prevents hyperglycemia, alveolar bone loss, and inflammation in a rat model of diabetes and periodontitis. J Periodontol. (2023) 94:376–88. doi: 10.1002/JPER.22-0262, PMID: 36322996

[88] Elsadek MF Ahmed BM Alkhawtani DM Zia SA. A comparative clinical, microbiological and glycemic analysis of photodynamic therapy and *Lactobacillus reuteri* in the treatment of chronic periodontitis in type-2 diabetes mellitus patients. Photodiagn Photodyn Ther. (2020) 29:101629. doi: 10.1016/j.pdpdt.2019.101629, PMID: 31870899

[89] Lu M Zhang Y Yuan X Zhang Y Zhou M Zhang T. Increased serum alpha-tocopherol acetate mediated by gut microbiota ameliorates alveolar bone loss through the STAT3 signalling pathway in diabetic periodontitis. J Clin Periodontol. (2023) 50:1539–52. doi: 10.1111/jcpe.13862, PMID: 37596824

[90] Malyshev ME Iordanishvili AK Prisyazhnyuk OV Bumai AO. The effect of probiotics on the secretory immunity of saliva in patients with type 2 diabetes. Stomatologiia. (2019) 98:26–9. doi: 10.17116/stomat20199806126, PMID: 31922506

[91] Bauer PV Duca FA Waise TMZ Dranse HJ Rasmussen BA Puri A. *Lactobacillus gasseri* in the upper small intestine impacts an ACSL3-dependent fatty acid-sensing pathway regulating whole-body glucose homeostasis. Cell Metab. (2018) 27:572–587.e6. doi: 10.1016/j.cmet.2018.01.013, PMID: 29514066

[92] Feizollahzadeh S Ghiasvand R Rezaei A Khanahmad H Sadeghi A Hariri M. Effect of probiotic soy Milk on serum levels of adiponectin, inflammatory mediators, lipid profile, and fasting blood glucose among patients with type II diabetes mellitus. Probiot Antimicrob Prot. (2017) 9:41–7. doi: 10.1007/s12602-016-9233-y, PMID: 27757829

[93] Hatakka K Martio J Korpela M Herranen M Poussa T Laasanen T. Effects of probiotic therapy on the activity and activation of mild rheumatoid arthritis--a pilot study. Scand J Rheumatol. (2003) 32:211–5. doi: 10.1080/03009740310003695, PMID: 14626627

